# Paired rRNA-depleted and polyA-selected RNA sequencing data and supporting multi-omics data from human T cells

**DOI:** 10.1038/s41597-020-00719-4

**Published:** 2020-11-09

**Authors:** Li Chen, Ruirui Yang, Tony Kwan, Chao Tang, Stephen Watt, Yiming Zhang, Guillaume Bourque, Bing Ge, Kate Downes, Mattia Frontini, Willem H. Ouwehand, Jing-wen Lin, Nicole Soranzo, Tomi Pastinen, Lu Chen

**Affiliations:** 1 Key Laboratory of Birth Defects and Related Diseases of Women and Children of MOE, Department of Laboratory Medicine, State Key Laboratory of Biotherapy, West China Second Hospital, Sichuan University, Chengdu, Sichuan 610041 China; 2 Human Genetics, McGill University, 740 Dr. Penfield, Montreal, QC H3A 0G1 Canada; 3 Department of Human Genetics, The Wellcome Trust Sanger Institute, Wellcome Trust Genome Campus, Hinxton, Cambridge CB10 1HH UK; 4 Department of Haematology, University of Cambridge, Cambridge Biomedical Campus, Long Road, Cambridge, CB2 0PT UK; 5 National Health Service (NHS) Blood and Transplant, Cambridge Biomedical Campus, Long Road, Cambridge, CB2 0PT UK; 6 East Midlands and East of England Genomic Laboratory Hub, Cambridge University Hospitals NHS Foundation Trust, Cambridge Biomedical Campus, Cambridge, UK; 7 Institute of Biomedical & Clinical Science, College of Medicine and Health, University of Exeter Medical School, RILD Building, Barrack Road, Exeter, EX2 5DW UK; 8 British Heart Foundation Centre of Excellence, Cambridge Biomedical Campus, Long Road, Cambridge, CB2 0PT UK; 9 British Heart Foundation Centre of Excellence, Division of Cardiovascular Medicine, Addenbrooke’s Hospital, Hills Road, Cambridge, CB2 0QQ UK; 10 The National Institute for Health Research Blood and Transplant Unit (NIHR BTRU) in Donor Health and Genomics at the University of Cambridge, Strangeways Research Laboratory, University of Cambridge, Wort’s Causeway, Cambridge, CB1 8RN UK; 11 Center for Pediatric Genomic Medicine, Children’s Mercy Kansas City, 2401 Gilham Rd., Kansas City, 64108 MO USA

**Keywords:** Data publication and archiving, RNA sequencing, Genetic variation

## Abstract

Both poly(A) enrichment and ribosomal RNA depletion are commonly used for RNA sequencing. Either has its advantages and disadvantages that may lead to biases in the downstream analyses. To better access these effects, we carried out both ribosomal RNA-depleted and poly(A)-selected RNA-seq for CD4^+^ T naive cells isolated from 40 healthy individuals from the Blueprint Project. For these 40 individuals, the genomic and epigenetic data were also available. This dataset offers a unique opportunity to understand how library construction influences differential gene expression, alternative splicing and molecular QTL (quantitative loci) analyses for human primary cells.

## Abstract (summary)

**Table:** 

Measurement(s)	RNA • gene expression
Technology Type(s)	RNA-seq of coding RNA • RNA-seq of total RNA • bioinformatics analysis
Factor Type(s)	gene expression level quantification • ribosomal RNA-depleted and poly(A)-selected RNA-seq • type of human primary cells
Sample Characteristic - Organism	Homo sapiens

Machine-accessible metadata file describing the reported data: 10.6084/m9.figshare.12932711

## Background & Summary

RNA sequencing (RNA-seq) that utilises next-generation sequencing (NGS) is a powerful tool to understand transcriptional diversity and regulation at bulk and single-cell level. Using RNA-seq, we not only can perform differential gene expression analysis with better resolution, but also comprehensively study alternative splicing, RNA editing and allele-specific expression, and all of which can be extended to investigate molecular quantitative trait loci (QTL) when genotypes are available at a population level.

In a eukaryotic cell, 80% of the total RNAs are ribosomal RNA (rRNA)^1,2^, whereas the other 5% is polyadenylated positive (poly(A)+) mRNA^3,4^. The two most commonly used selection methods, poly(A)-selected and rRNA-depleted (ribo-minus), selectively removes a distinct set of RNAs: poly(A) negative RNAs and rRNA, respectively. The poly(A)-selected protocol enriches poly(A) + transcripts including mRNAs and many non-coding RNAs^5,6^, and also reduces the amounts of pre-mRNAs. It has become a widely used RNA-seq protocol thanks to its low-noise rate. In contrast, rRNA-depleted removes cytoplasmic and mitochondrial rRNA and thus includes poly(A) + mRNA, as well as non-coding RNAs or protein-coding mRNAs that are not polyadenylated^7^. These two library construction protocols, sequencing different fractions of the transcriptome, may lead to complex technical bias. Indeed, it is reported that different RNA sample preparations may result in significant variations in the quantification of gene expression^5^. Furthermore, the influences introduced by these two protocols on splicing quantification, and molecular QTL analysis (such as expression QTL) are largely unknown. Currently, large population genetic studies, such as GTEx^8^, lymphoblastoid cell lines (LCL) from the 1000 genome and HapMap project^9^ mostly used poly(A)-selected protocol, therefore lacking the information of non-poly(A)-transcripts. In this study, we constructed both rRNA-depleted and poly(A)-selected RNA-seq libraries for CD4 +T cells from 40 donors in the Blueprint project^10^. This dataset informs understanding of how these two library construction methods affect downstream analyses, and helps develop bioinformatic tools to avoid or reduce artefacts that were introduced during experimental procedures.

## Methods

### Human subjects and sample collection

**Fig. 1:**
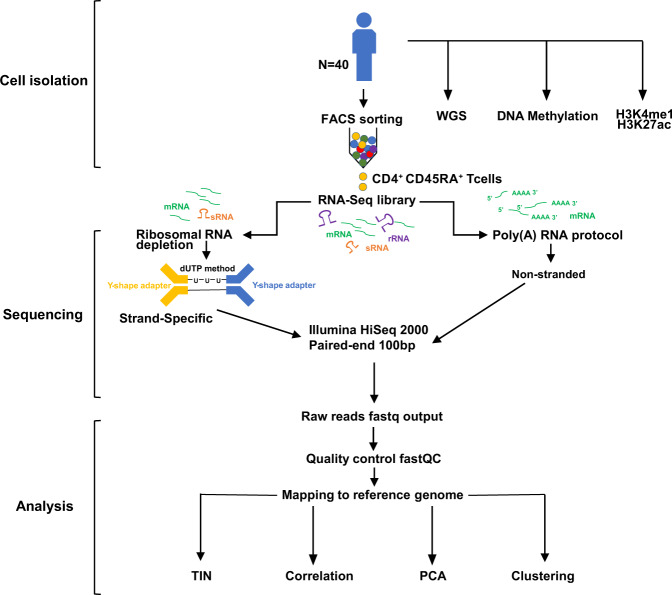
Study design of paired poly(A)-selected and ribosomal RNA-depleted RNA-sequencing.

These methods are expanded versions of descriptions in our previously published studies^10^. As described previously, blood was obtained from donors who were members of the NIHR Cambridge BioResource (http://www.cambridgebioresource.org.uk/) with informed consent (REC 12/EE/0040) at the NHS Blood and Transplant, Cambridge. The schematic for sample collection and processes were shown in Fig. 1. A unit of whole blood (475 mL) was collected in 3.2% Sodium Citrate. An aliquot of this sample was collected in EDTA for genomic DNA purification. A full blood count (FBC) for all donors was obtained from the EDTA blood sample, collected in parallel with the whole-blood unit, using a Sysmex Haematological analyser. The level of C-reactive protein (CRP), an inflammatory marker, was also measured in the sera of all individuals. All donors recruited in this study had FBC and CRP parameters within the normal healthy range. Blood was processed within 4 hr of collection.

### CD4^+^ T cell enrichment

Whole blood was diluted 1:1 in a buffer of Dulbecco’s Phosphate Buffered Saline (PBS, Sigma) containing 13 mM sodium citrate tribasic dehydrate (Sigma) and 0.2% human serum albumin (HSA, PAA) and separated using an isotonic Percoll gradient of 1.078 g/ml (Fisher Scientific). Peripheral blood mononuclear cells (PBMCs) were collected and washed twice with buffer, diluted to 25 million cells/mL and separated into two layers, a monocyte rich layer and a lymphocyte rich layer, using a Percoll gradient of 1.066 g/ml. Cells from each layer were washed in sampleemented PBS (13 mM sodium citrate and 0.2% HSA) and the subsets were purified using an antibody/magnetic bead strategy. CD4^+^ naive T cells were negatively selected using an EasySep Human Naive CD4^+^ T Cell Enrichment Kit (StemCell) according to the manufacturer’s instructions. The purity of each cell preparation was assessed by multicolor FACS using conjugated antibodies for CD4 (RPA-T4, BD) and CD45RA (HI100, BD) for naive CD4^+^ T cells. The purity of the cells was provided in Table 1.

**Table 1:** Summary of the cell purity, RNA quality and sequencing of poly(A)-selected RNA-seq. * indicates the sequencing depth of the rRNA-depleted samples.

Donor ID	Cell purity (FACS, %)	RIN	TIN (median)	rRNA-depleted reads (Million)*	Poly(A)-selected reads (Million)	Q30 (%)	GC (%)	Uniquely mapped reads (%)	Average mapped length
S000GZ	94.8	9.8	79.3	46.5	78.0	86.5	48.0	93.4	198.4
S000X1	97.2	9.1	79.3	61.4	64.8	86.1	48.0	93.6	198.5
S0010Q	95.5	9.3	79.4	54.3	51.9	86.5	48.0	93.3	198.4
S0012M	94.2	9.6	78.9	56.2	54.7	86.2	48.0	93.0	198.4
S001C2	94.3	9.6	79.1	36.8	57.1	88.5	48.0	93.8	198.5
S001GV	96.1	9.5	79.3	68.4	60.4	88.3	48.0	93.6	198.5
S001KN	95.7	9.8	79.5	69.0	58.7	88.4	48.0	94.1	198.6
S001NH	97.4	9.7	79.5	45.8	69.2	87.9	48.0	94.0	198.5
S001T5	95.7	9.6	79.3	31.9	60.6	86.8	48.0	93.6	198.4
S0021K	97.6	9.8	79.3	71.4	56.7	86.3	48.0	93.3	198.3
S0026A	97.7	9.7	79.6	53.6	61.7	86.4	48.0	93.6	198.3
S00294	94.9	9.8	79.0	57.4	63.7	86.6	47.0	92.6	198.1
S002EV	NA	9.6	78.0	49.2	19.9	83.2	47.0	92.8	198.3
S002FT	94.3	9.6	78.6	50.1	27.9	83.1	48.0	93.2	198.3
S002MF	96.1	9.6	78.7	51.7	33.4	83.1	48.0	93.4	198.3
S002WW	95.8	9.5	79.1	54.0	35.4	82.4	48.0	92.7	198.3
S002XU	95.9	9.1	78.8	51.5	36.3	83.4	47.0	92.2	198.3
S0031G	95.8	9.5	78.4	41.1	29.4	82.9	48.0	93.2	198.3
S0032E	98.2	9.3	77.9	54.7	32.9	83.3	47.0	92.6	198.4
S00382	98.6	9.6	78.5	62.1	32.1	84.2	47.0	92.9	198.4
S003AZ	99.1	9.5	78.5	68.6	34.1	84.5	47.0	92.8	198.4
S003JH	94.2	9.7	78.5	80.1	33.3	84.3	47.5	93.3	198.4
S003P5	95.1	9.5	77.9	48.7	24.2	81.7	48.0	92.9	198.3
S003Q3	95.0	9.9	77.9	53.2	27.6	81.8	48.0	92.8	198.3
S003R1	98.4	9.9	78.7	60.5	27.7	84.3	48.0	93.1	198.4
S0041C	95.0	9.5	78.7	76.7	27.5	84.3	48.0	93.4	198.4
S004M7	98.0	10.0	79.2	134.9	27.3	83.9	48.0	93.4	198.3
S004N5	93.5	9.7	78.8	71.1	30.8	84.2	48.0	93.6	198.4
S005N1	97.4	9.7	78.6	61.5	29.9	81.5	48.0	93.2	198.3
S005VM	93.5	NA	77.5	52.6	25.2	81.3	48.0	93.0	198.3
S005WK	96.8	9.0	77.6	63.0	24.5	81.6	48.0	93.2	198.4
S00630	89.9	9.5	77.8	52.8	22.4	81.5	47.0	93.4	198.3
S0064Z	96.9	9.9	78.3	57.4	27.5	81.7	47.0	93.3	198.4
S006XE	96.5	9.6	78.2	58.9	29.5	81.9	47.0	93.3	198.4
S007CF	99.0	9.0	77.4	59.4	23.9	88.7	46.0	93.4	198.5
S007DD	98.0	9.0	77.7	61.1	20.7	88.5	47.0	93.4	198.4
S007F9	98.2	9.0	78.4	70.6	35.2	87.8	48.0	93.2	198.3
S007G7	97.7	NA	78.2	67.7	30.4	88.5	47.0	93.5	198.5
S007PQ	91.9	8.8	77.6	91.9	30.0	88.5	46.0	93.2	198.4
S007VE	94.5	8.6	77.9	55.4	37.3	88.5	47.5	93.6	198.5

### RNA isolation and sequencing

Following purification, cells were lysed in TRIZOL reagent (Life Technologies) at a concentration of approximately 2.5 million cells/ml. RNA was extracted as per the manufacturer’s instructions, resuspended in ultra-pure water and quantified (Qubit, Invitrogen) prior to library preparation. The RIN (RNA integrity number) values were provided in Table 1. The same RNA samples were subjected to two RNA selection methods: TruSeq Stranded Total RNA Kit with Ribo-Zero Gold (Illumina) and TruSeq RNA Library Preparation Kit v2 (Illumina) for poly(A) + mRNA enrichment following manufacturer’s protocols (RS-122-9001DOC and RS-122-2001, respectively). Adaptor-ligated libraries were amplified and indexed via PCR. The 100 bp paired-end (PE) libraries were sequenced on Illumina’s HiSeq. 2000 instrument.

### Data processing

#### Quality control

The quality of the raw sequence data was checked using FastQC software (v0.11.8) (http://www.bioinformatics.babraham.ac.uk/projects/fastqc/) and RSeQC package (v3.0.1)^11^ (http://rseqc.sourceforge.net/).

#### Alignment

The paired-end reads were aligned to the human reference genome (GRCh37) using STAR (v2.7.1a)^12^ and HISAT2 (v2.1.0)^13^ with default parameters, using the Gencode version 19 annotation (https://www.gencodegenes.org/human/).

#### Aligned reads distribution

In RSeQC package^11^, the geneBody_coverage.py script was used for calculating the gene body coverage of the mapped reads; the read_distribution.py script was used to calculate how mapped reads were distributed over genome feature; the tin.py script was used to evaluate RNA integrity at the transcript level.

#### Gene expression quantification

The gene expression and “ExonOnly” expression level which only includes exonic reads were quantified using the HTSeq (v0.11.2)^14^, respectively. The parameter of “-s reverse” was set for the strand-specific rRNA-depleted samples, whereas “-s no” for the non-strand specific poly(A)-selected samples. The raw read counts were then normalized by their library size factors and were regularized-logarithm (rlog) transformed to stabilize the variance across the samples using DESeq2 (v1.28.1)^15^. Pearson’s correlation coefficients were calculated for each gene between the paired RNA-seq samples from the same individual. For unsupervised clustering analysis, we required that a gene has more than 10 reads in 20% of the samples. These quantification files were stored in public repository^16^.

#### Batch effect correction

The gene expression quantifications were corrected for batches using ComBat from the sva R package (v3.36.0)^17^.

## Data Records

The raw fastq files and aligned BAM files for the paired RNA-seq in naive CD4 + T cells from 40 individuals were deposited at Synapse^18^. The multi-omics processed files, including the sample information, the quantifications of gene expression, isoform, exonOnly (before and after ComBat^17^), splicing junction from both protocols, the genotype from WGS, the quantification of DNA methylation and Chip-seq of two histone markers (H3K4me1 and H3K27ac) of the corresponding individuals, were uploaded in figshare^16^. The other multi-omics files in three major human immune cell types (CD14+ monocytes, CD16+ neutrophils, and naive CD4+ T cells) from up to 197 individuals were published previously^10^, including the additional 132 rRNA-depleted RNA-seq for naive CD4+ T cells and genotypes from WGS at European Genome-phenome Archive (EGA) under accession numbers EGAD00001002671^19^ and EGAD00001002663^20^, respectively.

## Technical Validation

### Cell purity, RNA integrity and sequencing quality

We used EasySep Human Naive CD4+ T Cell Enrichment Kit for cell enrichment and achieved an average purity of 96% as assessed by multicolor FACS (Table 1). All RNA samples used for library construction had RNA integrity (RIN) values over 8.6 (Table 1). Moreover, the RNA integrity at transcript level was further evaluated using the Transcript Integrity Number (TIN) algorithm, calculated using the tin.py script from the RSeQC package^11^. TIN calculates a score ranging from 0 to 100 for each expressed transcript, and the medTIN (median TIN score across all the transcripts) can be used to measure the RNA integrity at the sample level. All the poly(A)-selected samples have TIN scores above 77, with a mean = 78.58 and SD = 0.64 (Table 1). We constructed both ribosomal RNA depleted and poly(A)-selected RNA-seq libraries for 40 CD4^+^ naïve T cells (Fig. 1 and Table 1).

**Fig. 2:**
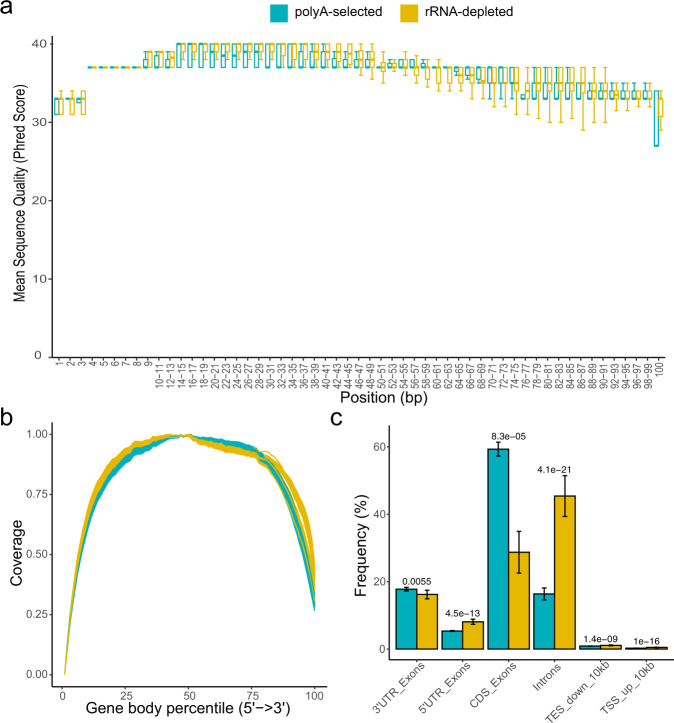
Summary of key quality control metrics. (**a**) Boxplot of average sequence quality per base per sample. Blue boxes indicate data gathered using poly(A)-selected and yellow boxes indicate rRNA-depleted. (**b**) Reads distribution along the gene body. Relative coverage of uniquely mapped tags generated based on the poly(A)-selected and rRNA-depleted RNA-seq. (**c**) Frequency of counts in various gene regions. The statistical test is Student’s t-test, and the error bars depict the standard deviation.

The average of sequencing depth in poly(A)-selected and rRNA-depleted libraries was 38.8 M (SD = 15.93) and 60.3 M (SD = 16.7), respectively. The quality of sequencing per base was assessed using FastQC, and the Q30 is over 81.3%. There is no significant difference in the distribution of average quality score per base between the poly(A)-selected and rRNA-depleted libraries (Fig. 2a). The reads generated using both methods were distributed uniformly across the gene body (Fig. 2b), indicating the high integrity of RNA samples and no obvious 3’ bias. The uniquely mapped reads account for over 92% of all reads in all samples, indicating the high quality of sequencing (Table 1). The quality, sequencing depth and alignment statistics of all RNA samples are shown in Table 1. We further compared the gene regions that the reads of the two protocols mapped to, and found that the poly(A)-selected RNA-seq has a significantly higher level of reads aligned to coding and exonic regions, but has a much lower level in intronic regions (Fig. 2c), consistent with the observation of the previous reports^1,5^.

### Gene expression quantification

**Fig. 3:**
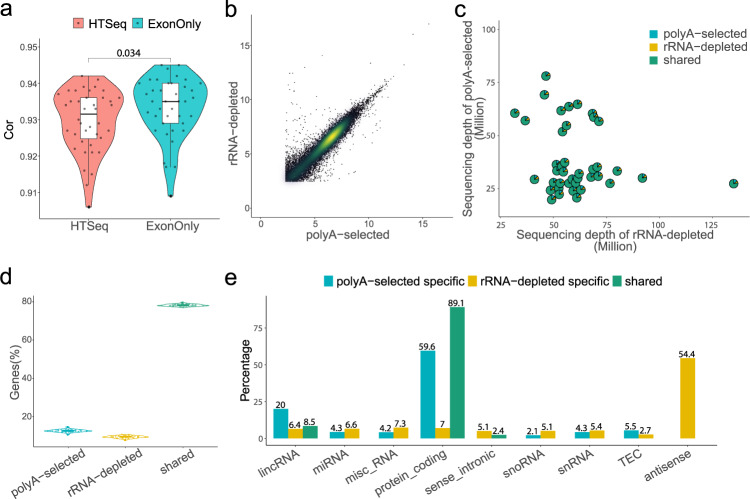
Comparison of gene expression identification between poly(A)-selected and rRNA-depleted. (**a**) Pearson’s correlation coefficients of the gene expression between 40 paired samples using HTSeq and ExonOnly quantification using STAR. (**b**) Scatter plot of rlog gene expression in poly(A)-selected RNA-seq and rRNA-depleted RNA-seq of one sample. (**c**) Scatterpie plot of paired genes identified in both datasets using ExonOnly. The x-axis and y-axis showed the sequencing depth of rRNA-depleted and poly(A)-selected of each pair respectively, and the pie chart illustrates the fractions of shared and protocol-specific genes. (**d**) Violin plot showing the percentage of genes that are library-specific and shared between two sequencing libraries. (**e**) Percentage of each biotype of library-specific and shared genes, biotypes accounting for more than 2% of all genes were shown (pseudogenes and antisense genes from non-stranded poly(A)-selected samples were excluded).

To assess the gene expression profile from poly(A)-selected and rRNA-depleted RNA-seq data, we quantified gene expression based on both STAR^12^ and HISAT2^13^ aligners, respectively. For these 40 paired samples, the average correlation coefficient is over 0.94, with a relatively higher correlation using STAR aligner (mean correlation coefficient increases by 0.006, Wilcoxon test, p = 0.0049). When only concerned exonic reads (ExonOnly) using STAR aligner, the correlation coefficients were further improved (Wilcoxon test, p = 0.034) (Fig. 3a), indicating the intronic reads affect the correlation between the paired samples. The correlation was also illustrated in a scatter plot showing paired poly(A)-selected and rRNA-depleted RNA-seq from one of 40 pairs (Fig. 3b), suggesting gene expression between two library protocols are highly correlated with some library-specific expressed genes.

Next, we compared the number and biotypes of the detected gene from both sequencing libraries after filtering the lowly expressed genes (log10 normalized expression less than 1). While on average 78.21% of genes were shared, 12.5% and 9.3% of genes were specific in poly(A)-selected and rRNA-depleted library, respectively. We found that these percentages of shared and library-protocol-specific genes were with little variances (SD ranging from 0.55% to 0.67%) in the difference of sequencing depth (Fig. 3c,d), indicating that our sequencing depths may reach saturation of quantification of the genes expressed in human CD4+ T cells in both library protocols. As expected, the poly(A)-selected protocol tends to identify more protein-coding genes, whereas the rRNA-depleted protocol, given it can identify non-poly(A) genes and has sequence strand information, identify more genes that are misc-RNA, snoRNA and antisense (Fig. 3e).

### Unsupervised clustering analysis

**Fig. 4:**
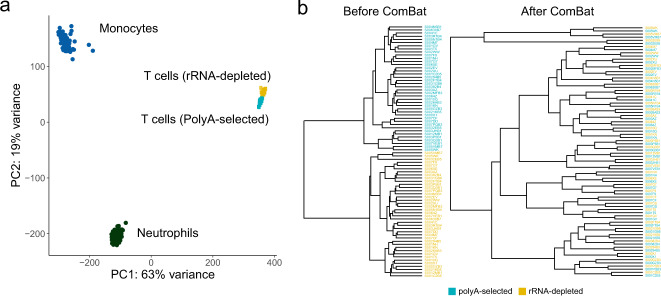
Unsupervised clustering analysis. (**a**) PCA of three cell types in the Blueprint project. (**b**) Hierarchical clustering of ExonOnly quantification before (left) and after (right) batch correction using ComBat based on STAR alignment.

Principal component analysis (PCA) of the data profiles in the three cell types (monocyte (n = 196), neutrophil (n = 197), CD4 + (rRNA-depleted n = 40, poly(A)-selected n = 40)) of the Blueprint project revealed that samples from the same cell type clustered closely, regardless of library construction methods (Fig. 4a). Next, we clustered the expression levels of all genes of the 40 paired CD4+ T cells using ExonOnly quantification based on STAR aligner (Fig. 4b). The samples from the two library methods were gathered together, reflecting the batch effects due to the library construction. After applying ComBat^17^, the 40 paired samples from different library construction methods were all clustered together (Fig. 4b), indicating that the improved expression quantification method and an effective batch correction can minimize the biases introduced in library construction.

## Usage Notes

In general, the rRNA-depleted protocol captures different RNA species and is more efficient in quantifying linear non-poly(A) transcripts and circular RNAs. Therefore when the researchers are interested in analysing them, rRNA-depleted protocol is the one to use. However, if the protein-coding genes are the primary research targets, the poly(A)-selected protocol can identify more genes using the same sequencing depth and yield much less intronic reads. Nevertheless, our results showed that the shared genes between two protocols are highly correlated after applying ExonOnly quantification and batch correction.

Using these 40 paired poly(A)-selected and rRNA-depleted RNA-seq data from naive CD4+ T cell, the effects of library construction on the quantification of gene expression, alternative splicing and RNA editing can be assessed. One can also use this dataset to test the quantification or batch correction methods which can minimize the biases caused by library protocols. Furthermore, the 40 individuals enrolled in Blueprint Project^10^ have comprehensive information including WGS, DNA methylation and Chip-seq of two histone markers (H3K4me1 and H3K27ac). This enables further investigation of the effect of library construction on multi-omics integration analysis and population genetics such as molecular QTLs of expression, splicing and RNA editing.

## Supplementary information

Sample Information

## Data Availability

The codes used in this article were deposited in https://github.com/LuChenLab/40Tcells.
