# Supplementary material for: Paired rRNA-depleted and polyA-selected RNA sequencing data and supporting multi-omics data from human T cells
Source: Sci Data. 2020 Nov 9;7:376. doi: 10.1038/s41597-020-00719-4 (PMC7652884; doi:10.1038/s41597-020-00719-4)
Supplement: Supplementary file 1 — Sample Information [file 41597_2020_719_MOESM1_ESM.pdf]

EGA ID	SAMPLE ID	DONOR_ID	DONOR_SEX	DONOR_AGE	LIBRARY PROTOCOL
EGAR00001193011	S00382B4_mRNA	S00382	Male	60 - 65	polyA-selected
EGAR00001193012	S00382B4_RNA	S00382	Male	60 - 65	rRNA-depleted
EGAR00001193013	S00294B5_mRNA	S00294	Male	50 - 55	polyA-selected
EGAR00001193014	S00294B5_RNA	S00294	Male	50 - 55	rRNA-depleted
EGAR00001193015	S007PQB3_mRNA	S007PQ	Male	60 - 65	polyA-selected
EGAR00001193016	S007PQB3_RNA	S007PQ	Male	60 - 65	rRNA-depleted
EGAR00001193017	S003Q3B1_mRNA	S003Q3	Female	50 - 55	polyA-selected
EGAR00001193018	S003Q3B1_RNA	S003Q3	Female	50 - 55	rRNA-depleted
EGAR00001193019	S001C2B5_mRNA	S001C2	Male	65 - 70	polyA-selected
EGAR00001193020	S001C2B5_RNA	S001C2	Male	65 - 70	rRNA-depleted
EGAR00001193021	S001KN_mRNA	S001KN	Male	40 - 45	polyA-selected
EGAR00001193022	S001KN_RNA	S001KN	Male	40 - 45	rRNA-depleted
EGAR00001193023	S003JHB1_mRNA	S003JH	Female	65 - 70	polyA-selected
EGAR00001193024	S003JHB1_RNA	S003JH	Female	65 - 70	rRNA-depleted
EGAR00001193025	S005N1B4_mRNA	S005N1	Male	55 - 60	polyA-selected
EGAR00001193026	S005N1B4_RNA	S005N1	Male	55 - 60	rRNA-depleted
EGAR00001193027	S0031GB6_mRNA	S0031G	Male	65 - 70	polyA-selected
EGAR00001193028	S0031GB6_RNA	S0031G	Male	65 - 70	rRNA-depleted
EGAR00001193029	S005VMB7_mRNA	S005VM	Male	55 - 60	polyA-selected
EGAR00001193030	S005VMB7_RNA	S005VM	Male	55 - 60	rRNA-depleted
EGAR00001193031	S007F9_mRNA	S007F9	Female	65 - 70	polyA-selected
EGAR00001193032	S007F9_RNA	S007F9	Female	65 - 70	rRNA-depleted
EGAR00001193033	S002MFB3_mRNA	S002MF	Male	65 - 70	polyA-selected
EGAR00001193034	S002MFB3_RNA	S002MF	Male	65 - 70	rRNA-depleted
EGAR00001193035	S002FTB4_mRNA	S002FT	Male	60 - 65	polyA-selected
EGAR00001193036	S002FTB4_RNA	S002FT	Male	60 - 65	rRNA-depleted
EGAR00001193037	S004M7_mRNA	S004M7	Female	60 - 65	polyA-selected
EGAR00001193038	S004M7_RNA	S004M7	Female	60 - 65	rRNA-depleted
EGAR00001193039	S000GZB3_mRNA	S000GZ	Male	50 - 55	polyA-selected
EGAR00001193040	S000GZB3_RNA	S000GZ	Male	50 - 55	rRNA-depleted
EGAR00001193041	S007CF_mRNA	S007CF	Female	60 - 65	polyA-selected
EGAR00001193042	S007CF_RNA	S007CF	Female	60 - 65	rRNA-depleted
EGAR00001193043	S001GV_mRNA	S001GV	Female	60 - 65	polyA-selected
EGAR00001193044	S001GV_RNA	S001GV	Female	60 - 65	rRNA-depleted
EGAR00001193045	S004N5B1_mRNA	S004N5	Male	55 - 60	polyA-selected
EGAR00001193046	S004N5B1_RNA	S004N5	Male	55 - 60	rRNA-depleted
EGAR00001193047	S0064Z_mRNA	S0064Z	Male	50 - 55	polyA-selected
EGAR00001193048	S0064Z_RNA	S0064Z	Male	50 - 55	rRNA-depleted
EGAR00001193049	S002WW_mRNA	S002WW	Male	45 - 50	polyA-selected
EGAR00001193050	S002WW_RNA	S002WW	Male	45 - 50	rRNA-depleted
EGAR00001193051	S003R1B4_mRNA	S003R1	Female	45 - 50	polyA-selected
EGAR00001193052	S003R1B4_RNA	S003R1	Female	45 - 50	rRNA-depleted
EGAR00001193053	S007DD_mRNA	S007DD	Female	50 - 55	polyA-selected
EGAR00001193054	S007DD_RNA	S007DD	Female	50 - 55	rRNA-depleted
EGAR00001193055	S001NH_mRNA	S001NH	Male	55 - 60	polyA-selected
EGAR00001193056	S001NH_RNA	S001NH	Male	55 - 60	rRNA-depleted
EGAR00001193057	S007VEB1_mRNA	S007VE	Male	30 - 35	polyA-selected
EGAR00001193058	S007VEB1_RNA	S007VE	Male	30 - 35	rRNA-depleted
EGAR00001193059	S00630B7_mRNA	S00630	Male	40 - 45	polyA-selected
EGAR00001193060	S00630B7_RNA	S00630	Male	40 - 45	rRNA-depleted
EGAR00001193061	S002XU_mRNA	S002XU	Male	45 - 50	polyA-selected

EGAR00001193062	S002XU_RNA	S002XU	Male	45 - 50	rRNA-depleted
EGAR00001193063	S0032EB5_mRNA	S0032E	Male	60 - 65	polyA-selected
EGAR00001193064	S0032EB5_RNA	S0032E	Male	60 - 65	rRNA-depleted
EGAR00001193065	S005WK_mRNA	S005WK	Male	65 - 70	polyA-selected
EGAR00001193066	S005WK_RNA	S005WK	Male	65 - 70	rRNA-depleted
EGAR00001193067	S0012MB1_mRNA	S0012M	Female	35 - 40	polyA-selected
EGAR00001193068	S0012MB1_RNA	S0012M	Female	35 - 40	rRNA-depleted
EGAR00001193069	S003P5B1_mRNA	S003P5	Male	45 - 50	polyA-selected
EGAR00001193070	S003P5B1_RNA	S003P5	Male	45 - 50	rRNA-depleted
EGAR00001193071	S002EV_mRNA	S002EV	Male	45 - 50	polyA-selected
EGAR00001193072	S002EV_RNA	S002EV	Male	45 - 50	rRNA-depleted
EGAR00001193073	S001T5_mRNA	S001T5	Male	50 - 55	polyA-selected
EGAR00001193074	S001T5_RNA	S001T5	Male	50 - 55	rRNA-depleted
EGAR00001193075	S0041C_mRNA	S0041C	Female	60 - 65	polyA-selected
EGAR00001193076	S0041C_RNA	S0041C	Female	60 - 65	rRNA-depleted
EGAR00001193077	S007G7_mRNA	S007G7	Male	60 - 65	polyA-selected
EGAR00001193078	S007G7_RNA	S007G7	Male	60 - 65	rRNA-depleted
EGAR00001193079	S0026AB3_mRNA	S0026A	Female	55 - 60	polyA-selected
EGAR00001193080	S0026AB3_RNA	S0026A	Female	55 - 60	rRNA-depleted
EGAR00001193081	S0021KB5_mRNA	S0021K	Male	35 - 40	polyA-selected
EGAR00001193082	S0021KB5_RNA	S0021K	Male	35 - 40	rRNA-depleted
EGAR00001193083	S003AZ_mRNA	S003AZ	Male	65 - 70	polyA-selected
EGAR00001193084	S003AZ_RNA	S003AZ	Male	65 - 70	rRNA-depleted
EGAR00001193085	S006XE_mRNA	S006XE	Male	65 - 70	polyA-selected
EGAR00001193086	S006XE_RNA	S006XE	Male	65 - 70	rRNA-depleted
EGAR00001193087	S0010Q_mRNA	S0010Q	Female	60 - 65	polyA-selected
EGAR00001193088	S0010Q_RNA	S0010Q	Female	60 - 65	rRNA-depleted
EGAR00001193089	S000X1_mRNA	S000X1	Female	50 - 55	polyA-selected
EGAR00001193090	S000X1_RNA	S000X1	Female	50 - 55	rRNA-depleted
